# The growing inadequacy of an open-ended Saffir–Simpson hurricane wind scale in a warming world

**DOI:** 10.1073/pnas.2308901121

**Published:** 2024-02-05

**Authors:** Michael F. Wehner, James P. Kossin

**Affiliations:** ^a^Applied Mathematics and Computational Research Division, Lawrence Berkeley National Laboratory, Berkeley, CA 94720; ^b^First Street Foundation, Brooklyn, NY 11201; ^c^Space Science and Engineering Center, University of Wisconsin–Madison, Madison, WI 11201

**Keywords:** tropical cyclone, climate change, Saffir-Simpson hurricane intensity scale

## Abstract

Global warming increases available sensible and latent heat energy, increasing the thermodynamic potential wind intensity of tropical cyclones (TCs). Supported by theory, observations, and modeling, this causes a shift in mean TC intensity, which tends to manifest most clearly at the greatest intensities. The Saffir–Simpson scale for categorizing damage based on the wind intensity of TCs was introduced in the early 1970s and remains the most commonly used metric for public communication of the level of wind hazard that a TC poses. Because the scale is open-ended and does not extend beyond category 5 (70 m/s windspeed or greater), the level of wind hazard conveyed by the scale remains constant regardless of how far the intensity extends beyond 70 m/s. This may be considered a weakness of the scale, particularly considering that the destructive potential of the wind increases exponentially. Here, we consider how this weakness becomes amplified in a warming world by elucidating the past and future increases of peak wind speeds in the most intense TCs. A simple extrapolation of the Saffir–Simpson scale is used to define a hypothetical category 6, and we describe the frequency of TCs, both past and projected under global warming, that would fall under this category. We find that a number of recent storms have already achieved this hypothetical category 6 intensity and based on multiple independent lines of evidence examining the highest simulated and potential peak wind speeds, more such storms are projected as the climate continues to warm.

## Significance

Global warming leads to more intense tropical cyclones (TCs). Three separate lines of evidence from both observations and models suggest that the open endedness of the 5th category of the Saffir–Simpson hurricane wind scale becomes increasingly problematic for conveying wind risk in a warming world. We investigate considering the extension to a 6th category of the Saffir–Simpson hurricane wind scale to communicate that climate change has caused the winds of the most intense TCs to become significantly higher.

The Saffir–Simpson hurricane wind scale is the most widely used metric to warn the public of the hazards of tropical cyclones (TCs) (1, 2). Introduced in the early 1970s by the United States National Hurricane Center (NHC) using estimates of peak wind, storm surge, and minimum central pressure to describe both wind- and water-driven destruction during TC landfall along a coast, it was altered in 2010 to be solely determined by 1-min-average maximum sustained winds at a height of 10 m. In this sense, the scale is used only to communicate risk from winds. Other TC hazards, such as precipitation, floods, and storm surge are communicated by the NHC via specialized metrics (see refs. 3–5). It is well understood that these water-related hazards are responsible for the largest share of TC-related mortality. For example, Rappaport (6) found that TC-related deaths in the United States were caused mostly by coastal storm surge (49%), followed by flooding from heavy rain (27%), while deaths caused directly by wind made up only 8% of total mortality. Still, TC wind hazard remains an important metric for communicating risk to the general public and is a critical metric when considering insured losses since many properties are insured against wind damage but not water damage.

The Saffir–Simpson scale is open-ended with category 5 storms of 70 m/s windspeed or greater. The open endedness of the scale is due largely to the observation at the time of the scale’s introduction that the combined effects of wind, surge, and rainfall in a category 5 impact would completely raze any structure. The later decision to reduce the scale to a wind-hazard-only scale to be complemented by other tools for communicating water-related hazards has somewhat skewed the original intent and underpinnings of the Saffir–Simpson scale. Our motivation here is to reconsider how the open-endedness of the scale can lead to an underestimation of risk, and, in particular, how this underestimation becomes increasingly problematic in a warming world.

Global warming has increased the energy available for TC intensification through increases in latent and sensible heat fluxes from warmer ocean temperatures (7). As a result, storm intensities well above the category-5 threshold are being realized and record wind speeds will likely continue to be broken as the planet continues to warm. In light of this, we introduce a hypothetical modification of the Saffir–Simpson hurricane wind scale to bound category 5 to peak wind speeds between 70 and 86 m/s and include an additional category 6 above that (Table 1). Previously, it has been argued that one particularly destructive storm, Typhoon Haiyan, should be included in a proposed category 6 (8), but Haiyan does not appear to be an isolated case. In this paper, we present three distinct motivations to consider that global warming increases the likelihood that other storms reach such intense wind speeds. The first is purely observational, that is, a number of recent storms have already reached our hypothetical category 6 wind speeds. The second is a formal detection and attribution analysis of increases in Emanuel’s Potential Intensity (PI) index (9, 10) revealing that the risk of these storms that reach our hypothetical category 6 has already been increased by human interference in the climate system. Climate model simulations of a warmer climate project further increases in this PI estimate of risk. The third is provided by analyzing projected changes in the most intense storms produced by multi-decadal simulations of high-resolution (~25 km) TC permitting global climate change models.

**Table 1.** Current and hypothetical hurricane wind scales

Tropical Depression	≤17 m/s
	≤38 mph
	≤33 kn
	≤62 km/h
Tropical Storm	18 to 32 m/s
	39 to 73 mph
	34 to 63 kn
	63 to 118 km/h
1 hurricane	33 to 42 m/s
	74 to 95 mph
	64 to 82 kn
	119 to 153 km/h
2	43 to 49 m/s
	96 to 110 mph
	83 to 95 kn
	154 to 177 km/h
3 major hurricane	50 to 58 m/s
	111 to 129 mph
	96 to 112 kn
	178 to 208 km/h
4	58 to 70 m/s
	130 to 156 mph
	113 to 136 kn
	209 to 251 km/h
5 (current)	>70 m/s
	>157 mph
	137 kn
	>252 km/h
5 (proposed)	70 to 86 m/s
	157 to 192 mph
	137 to 167 kn
	252 to 309 km/h
6 (proposed)	>86 m/s
	>192 mph
	>167 kn
	>309 km/h

## 1. Recent Extreme Maximum Wind Speeds in Observed Storms

**Fig. 1.**
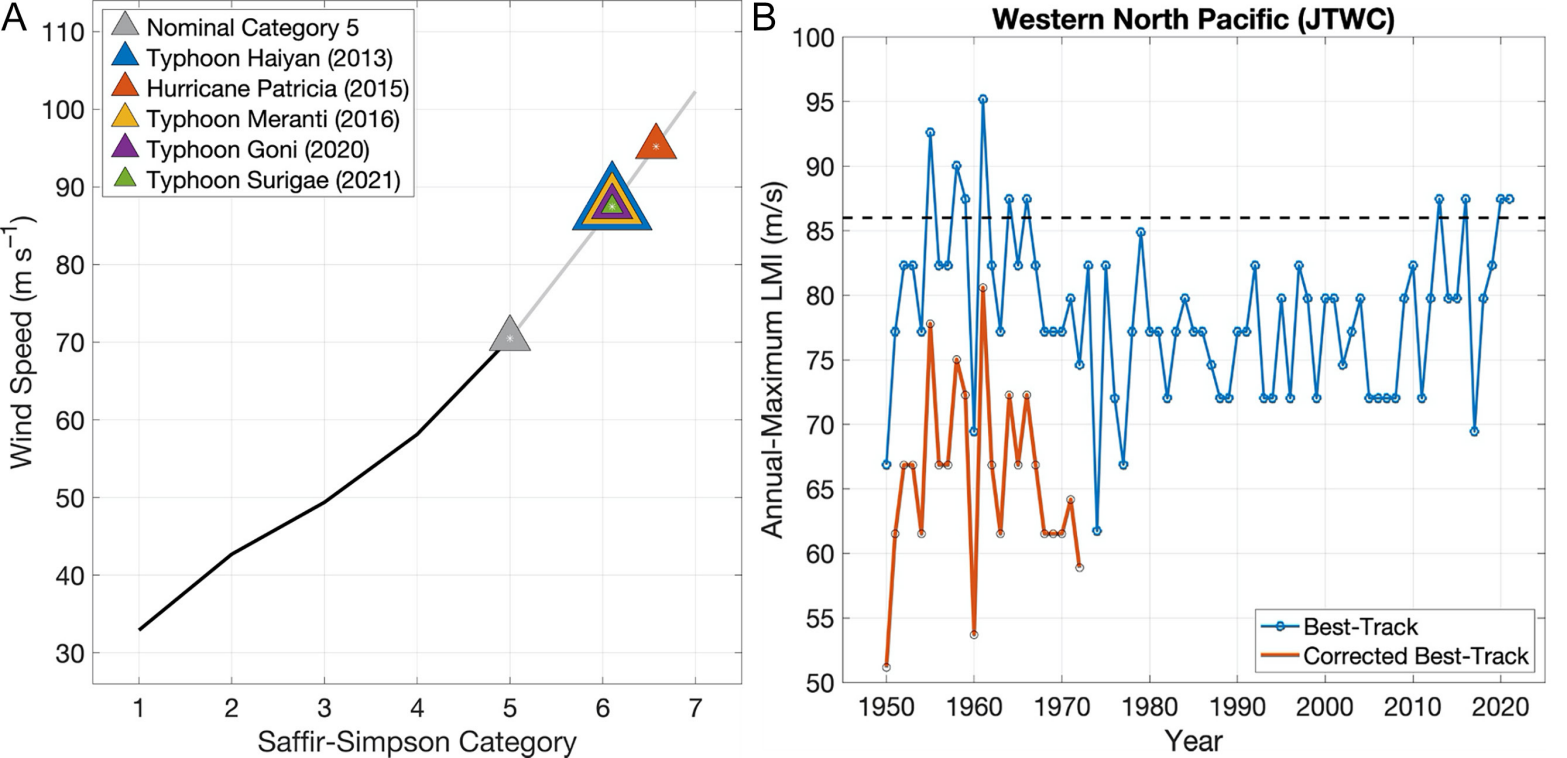
(*A*) The five recent storms that reached our hypothetical category 6 wind intensity. The triangles denote which storms reached these intensities. Since windspeeds are recorded in discrete 5 kn bins, the triangles overlap at those shared LMIs. (*B*) Time series of the annual-maximum LMI in the western North Pacific historical (IBTracs) record of intensity (blue), and the bias-corrected data prior to 1973 (red). The black dashed line identifies our hypothetical nominal category 6 intensity.

The 6th Assessment Report of the Intergovernmental Panel on Climate Change (IPCC AR6 WG1) found that “It is likely that the global proportion of categories 3 to 5 TC instances and the frequency of rapid intensification events have increased globally over the past 40 years” (11). Indeed, of the 197 TCs that were classified as category 5 during the 42-y period 1980 to 2021, which comprises the period of highest quality and most consistent data, half of them occurred in the last 17 y of the period (12). Five of those storms exceeded our hypothetical category 6 and all of these occurred in the last 9 y of the record. The most intense of these hypothetical category 6 storms, Patricia, occurred in the Eastern Pacific making landfall in Jalisco, Mexico, as a category 4 storm. The remaining category 6 storms all occurred in the Western Pacific. Two of them, Haiyan and Goni, made landfall on heavily populated islands of the Philippines. Haiyan was the costliest Philippines storm and the deadliest since the 19th century, long before any significant warning systems. Indeed, it has been argued (8) that Haiyan should be labeled category 6 and that its destructive potential by wind damage far exceeded a nominal category 5 storm. Meranti tracked between the main Philippine islands and Taiwan, causing damages in both nations before making its main landfall in eastern China causing severe inland flooding. Fig. 1*A* shows these 5 storms on the existing Hurricane Wind Scale and our proposed extension.

The historical records of TC intensities have several associated uncertainties (12, 13) that reduce confidence in past trends. In the western North Pacific, where all but one of the TCs shown in Fig. 1 occurred, intensities prior to 1973 have been shown to have a substantial-high bias, largely due to operational analysis methods of that time. A bias correction has been proposed by Emanuel (14) and discussed further in Landsea (15) and Emanuel (16). With full acknowledgment of the uncertainties, it is informative to look further back in the western North Pacific TC records to the beginning of the post-World War II aircraft reconnaissance period (Fig. 1*B*). When the post-World War II data are bias-corrected, the recent cluster of TCs that exceeded 86 m/s is unprecedented (Fig. 1*B*). Kossin et al. (17) demonstrated that positive trends in quantiles of the distribution of IBTracs Lifetime Maximum Intensity (LMI) over the 1982 to 2009 period were significant in higher quantiles but were substantially reduced in a corrected satellite dataset. In that analysis, the 95th percentile showed negative trends. However, all of the category 6 storms in the corrected IBTracs data (Fig. 1*B*) occur after 2009 and could alter that finding.

## 2. Detection, Attribution, and Projection of Changes in PI

Emanuel (9, 10) considered an idealized TC as a heat engine transporting thermal energy from the warm surface to cool storm top while producing the kinetic energy of the storm winds. This thermodynamical model of a TC can be idealized as a Carnot engine transporting energy from the ocean surface to the outflow level near the tropopause from which Emanuel derived his PI index. Sobel et al. (18) showed that positive simulated mean PI trends due to increasing greenhouse gases were nearly entirely offset by negative trends due anthropogenic sulfate aerosol emissions in the Northern Hemisphere over the 1850 to 2005 period in the Coupled Model Intercomparison Project (CMIP5) models. They note that simulated mean PI increases after about 1980 because of reductions in aerosol emissions principally due to clean air legislations.

As part of our case for adding a hypothetical category 6 at 85 m/s to the Saffir–Simpson hurricane wind scale, we take a different approach than Sobel et al. (18) by developing a detection and attribution analysis of changes in the extreme tail of the daily PI distribution as opposed to changes in its annual mean. As Sobel et al. (18) point out, change in the observed LMI distribution is one-sided and mainly in its higher quantiles as would be expected from a simple shift in the PI distribution. They also demonstrated that annual mean PI changes are a complicated function of the state of the atmosphere. Thus, similar to changes in the surface air temperature (19), one should not presume that changes in the tail of PI distribution are the same as its mean changes.

**Fig. 2.**
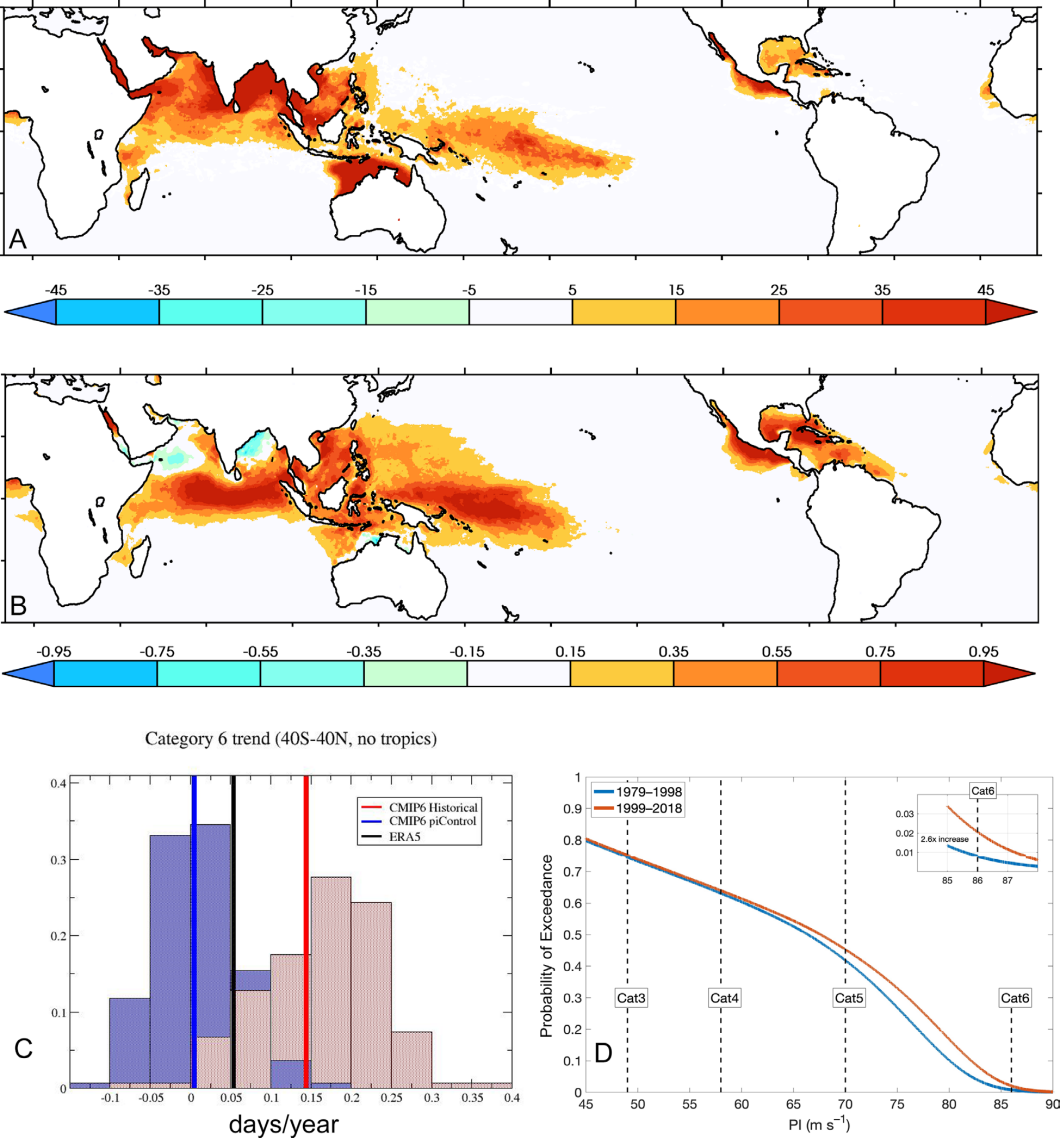
(*A*) ERA5 1979 to 2019 average annual exceedance of category 6 wind speed threshold. Units: days. (*B*) Linear rate of change in ERA5 average annual exceedance of category 6 wind speed threshold from 1979 to 2019. Units: days per year. (*C*) Linear trends in average annual exceedance of the category 6 wind speed threshold from 1979 to 2019 averaged over 40S to 40N excluding 10S to 10N. blue: CMIP6 *historical*, red: CMIP6 *piControl*, black: ERA5 reanalysis. (*D*) Increase in the tail of the PI distribution indicating a 2.6× increase in the chances of PI exceeding category 6 from the first half of the ERA5 reanalysis to its second half over the region 40S to 40N excluding 10S to 10N.

As a proxy for observations, we calculated PI from daily output fields of the most recent (20) reanalysis product of European Centre for Medium Range Weather Forecasting (ERA5) over the period from 1979 to 2019. We then calculated the annual number of days that PI exceeded the hypothetical category 6 threshold at each ocean grid point from 40S to 40N (Fig. 2*A*) and its linear trend (days/year) over that period (Fig. 2*B*). A convenient climate change detection variable is defined as the spatial average of this annual exceedance, excluding the region of 10S to 10N as intense TCs rarely occur there due to small Coriolis force. We conclude that a trend (Fig. 2*C*, black line) in this average Category 6 exceedance is detected because a Mann–Kendall statistical test confirms a positive trend with a very high degree of confidence (*P* < 0.01).

To make an attribution statement via Pearl causal inference (21), we then calculated daily PI from the *historical* and *piControl* experiments of the most recent version of the CMIP6 (22). Modeled input fields to the PI calculation were individually biased corrected using the ERA5 reanalysis as described in the *Materials and Methods* section. A two-sided student t test reveals that the *historical* and *piControl* ensemble mean trends averaged over this region are drawn from different distributions at a very high confidence level (*P* ≪ 0.01). Hence, the detected change in the ERA5 proxy for an observed PI change from 1979 to 2014 averaged over the 40S to 10S, 10N to 40N region is attributable to the human and natural forcing factors included in CMIP6 *historical* experiments. While we cannot formally rule out the influence of solar and volcanic forcing due to limited daily data available from the CMIP6 *hist-nat* experiments, previous attribution studies of global surface temperature, air temperature aloft, and specific humidity over ocean have (23–25). Analysis of the tail of the full PI probability density distribution from the ERA5 reanalysis further reveals that the chances of PI exceeding the category 6 threshold at any gridpoint is nearly tripled in the 1999 to 2018 period compared to 1979 to 1998 (Fig. 2*D*). Thus, we conclude with high confidence that anthropogenic global warming has increased the global risk of category 6 TCs since 1979 as reflected by the PI index.

**Fig. 3.**
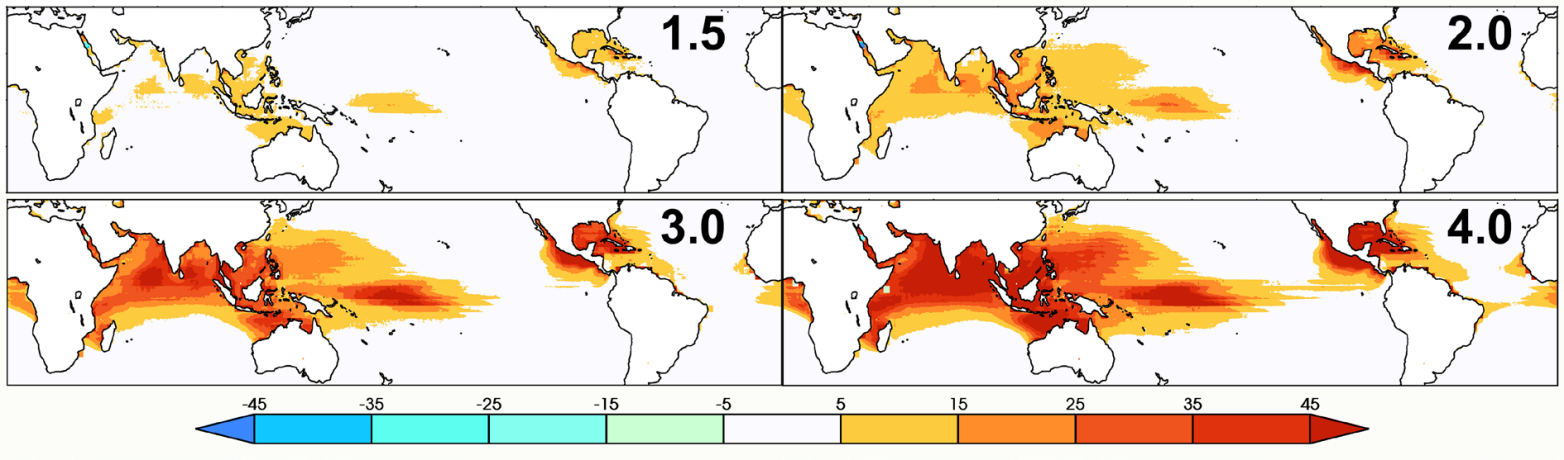
Change in annual PI exceedance of the category 6 threshold since present day at various future global warming levels from perturbed ERA5. *Upper* *Left*: 1.5 °C above preindustrial. *Upper* *Right*: 2.0 °C above preindustrial. *Lower* *Left*: 3.0 °C above preindustrial. *Lower* *Right*: 4.0 °C above preindustrial. Units: Days.

Despite the bias correction of the CMIP6 input to the PI calculation, most of the CMIP6 historical realizations exhibit a substantially higher trend in the annual exceedance of category 6 wind speed threshold than the ERA5 reanalysis. Hence, the CMIP6 future simulations exhibit very large increases in this exceedance as the climate warms. For a more conservative estimate of a future change, we borrowed a technique from pseudo-global warming simulations (26, 27) to create plausible future warmer conditions by perturbing the ERA5 reanalysis fields used as input to the PI code in Fig. 2 by changes calculated from an ensemble of CMIP6 SSP585 simulations (28). Fig. 3 shows the resulting change in the annual number of days where PI exceeds the category 6 wind speed threshold at global warming levels of 1.5, 2.0, 3.0, and 4.0 °C above preindustrial relative to the recent historical period (1979 to 2014).

Considering the density of observed intense TCs in products such as IBTracs (12), Fig. 2*A* reveals that the Philippines, parts of Southeast Asia and the Gulf of Mexico are regions where the risk of a category 6 storm is currently of concern. This risk near the Philippines is increased by approximately 50% at 2 °C above preindustrial and doubled at 4 °C. Increased risk category 6 storms in the Gulf of Mexico increases even more, doubling at 2 °C above preindustrial and tripling at 4 °C. While the present and future exceedances of the category 6 wind speed threshold are substantially higher in the Northern Indian Ocean, and the seas north of Australia, intense TCs are not nearly as commonly observed in these regions as conditions other than warm seas are not favorable for rapid intensification. We conclude then that these regions are not at as high a risk for category 6 storms.

## 3. Changes in Maximum Wind Speeds of TC Permitting Climate Model Simulations

Recent advances in high-performance computing technologies have enabled multi-decadal simulations at “TC permitting” resolutions of ~25 km or finer (29, 30). Models of this class vary considerably in the realism of their simulated storms but some are able to produce category 5 storms with credible relationships between maximum wind speeds and minimum central pressures (31–33). Storm counts and track distributions also compare favorably with observations in some of the models. However, it is important to note that eyewalls, rapid intensification, and other characteristics of very intense storms are not reproduced at such resolutions.

Previous analyses of multi-decadal simulations of models in this class have revealed a positive shift in the distribution of TC maximum lifetime wind speeds as temperatures rise regardless of whether the model can achieve wind speeds in the higher categories (34, 35). Some but not all projections find that the annual global number of tropical storms of all intensities decreases in warmer climates (36). Projections of changes in the frequency of intense TCs (category 4 and above) are then a complicated function of the cyclogenesis frequency change, the increase in average peak wind speeds, and the amount of global warming. Hence, even projections of the sign of the future change in risk of intense TCs cannot be made with confidence (11).

However, as the risk of category 6 storms is presently near but not zero, any shift in the tail of the distribution of TC wind speeds over the threshold would increase that risk. Here, we examine global warming projections from three of the TC permitting global climate models that can produce category 5 wind speeds. Two of them are atmosphere-only simulations where future ocean temperatures are perturbed from observations and future changes from coarser coupled climate model simulations. One is a nudged coupled ocean–atmosphere model. However, none of these models are directly comparable as experimental setup details differ.

The finite volume 25-km version of Community Atmospheric Model (fvCAM5) produces a large (45%) reduction compared to present in tropical storm frequency in a stabilized climate 3 °C above preindustrial temperatures (37). While no wind speeds over the category 6 threshold are found in the historical simulation, the model projects the annual chances of a category 6 storm somewhere on the planet to be about 2% at the 1.5 °C global warming level, 7% at the 2 °C level and 10% at the 3 °C level despite the large decrease in overall cyclogenesis.

The MRI-AGCM3.2 model from Japan’s Meteorological Research Institute reduces tropical storm frequency by only 18% in an end of 21st-century Special Report Emissions Scenario (SRES) A1B (38) emissions scenario (~2.75 °C above preindustrial). At this global warming level, the MRI-AGCM3.2 projects about a 50% chance of a category 6 storm per year but only produced a single storm at that intensity in a historically forced simulation of 1979 to 2003.

At the end of the 21st century under the RCP4.5 (39, 40) emissions scenario (~2 °C above preindustrial), the HiFLOR model, developed at the Geophysical Fluid Dynamics Laboratory, produces 9% more tropical storms than present. Under present-day conditions, the HiFLOR simulates about a 25% annual chance of a single category 6 storm, which is higher than observed (Section 1). Under the RCP4.5 scenario, the HiFLOR projects a doubling of the chances of a category 6 storm at the middle of the 21st century (~1.6 °C above preindustrial) and a quadrupling at the end of the 21st century (~2 °C above preindustrial).

None of these high-resolution climate model projections should be taken too literally. Indeed, Davis (41) criticizes models of this resolution stating that they “should not produce a realistic number of category 4 and 5 storms.” Indeed, most of them produce too few storms at or above category 4 and winds in the most intense simulated storms are weaker than the maximum IBTracs winds when integrated under current climate conditions. However, we present them to demonstrate that the widely projected shift in the tail of the distribution of TC maximum wind speeds will very likely cause the actual distribution’s tail to extend well over the category 6 threshold.

## 4. Discussion

Anthropogenic global warming has already significantly increased surface ocean and tropospheric air temperatures in regions where TCs form and propagate. The resulting increases in available sensible and latent heat energy increases the thermodynamic potential wind intensity of these storms. Here, we introduced a hypothetical extension to the Saffir–Simpson hurricane wind scale to reflect that the most intense TCs are becoming more intense and will continue to do so as the climate continues to warm. This extension is supported by three lines of evidence illustrating that the most extreme observed, potential, and simulated peak wind speed have already increased due to global warming.

In Section 1, we show that several recent storms have already exceeded this hypothetical category 6 wind speed threshold of 86 m/s (Fig. 1). Prior to the satellite era, eight Western Pacific TCs in the IBTracs database reached category 6 intensities between 1955 and 1966. Regular aircraft reconnaissance in the western Pacific ended in 1987 (42) but transcribed aircraft data publicly exists only from 1946 to 1965 and 1978.^*^ However, correction for the known inconsistencies between current maximum wind speeds estimates and those prior to 1973 reveals that the maximum IBTracs wind speeds in the aircraft and satellite eras are incomparable. This suggests that the hypothetical extension is only relevant to tropical storms of the modern era. In Section 2, we discuss observed and projected future changes in the tail of the distribution of TC PI (14). From the ERA5 reanalysis, we find that in regions where intense TCs often occur, PI exceeded the category 6 threshold from a week up to a month per year averaged over the 1979 to 2015 period. However, this exceedance of the category 6 threshold by PI is also a mostly recent phenomena as revealed by an increase in the annual exceedance frequency by about a day every 2 or 3 y in these regions over this period. Overall, the chances of PI exceeding the category 6 threshold have more than doubled since 1979. While the CMIP6 models do not directly rule out forced natural causes, an increase in category 6 exceedance frequency is shown in the simulations with observed anthropogenic changes to be different from zero with a high degree of confidence. Furthermore, previous attribution studies of increases in surface temperature, lower tropospheric temperature, tropopause, and specific humidity together with decreases in stratospheric temperatures strongly support a human induced increase in extreme PI. Future simulations derived from the CMIP6 models project even greater increases in the annual exceedance of the category 6 threshold in the regions where intense TCs currently occur. As an important caveat, we note that stability issues might place a limit on the upper bound of highest achievable TC wind speeds (43) that are not included in the formulation of PI. Nonetheless, the recent occurrence of the category 6 storms discussed in Section 1 suggests that this limit, if it exists, has not yet been reached.

Finally, in Section 3, we show that multi-decadal simulations from three high-resolution global climate models capable of producing category 5 TCs under current climate conditions all exhibit category 6 storms under conditions warmer than today. Even under the relatively low global warming targets of the Paris Agreement, the increased chances of category 6 storms are substantial in these simulations. We note that limitations of these TC-permitting models are important but that the bias of such models is generally for the most intense storms to be too weak due to resolution limitations. Hence, the risk implied by these simulations is likely underestimated.

These three independent lines of evidence for an increase in the intensity of the most intense TCs are entirely consistent with the theoretical expectation that more available energy is reflected in stronger TC wind speeds. The sociological aspects of the messaging embodied in public TC warnings (3, 44–47) are complicated, and despite its deficiencies in communicating the full range of TC risks, it seems unlikely that usage of the Saffir–Simpson scale will end anytime soon. Indeed, even when communicated with other measures of risk, the Saffir–Simpson scale appears to be that heard most loudly (47). Our results are not meant to propose changes to this scale, but rather to raise awareness that the wind-hazard risk from storms presently designated as category 5 has increased and will continue to increase under climate change. Warnings of TC risk must come from operational government centers in order to have credibility. TC risk messaging is currently a very active topic and changes in messaging are widely believed necessary to better inform the public about inland flooding and storm surge, phenomena that a wind-based scale is only tangentially relevant to. While adding a 6th category to the Saffir–Simpson Hurricane Wind Scale would not solve that issue, it could raise awareness about the perils of the increased risk of major TCs due to global warming. Clearly, to maximize effectiveness, detailed sociological research (3, 46, 48, 49) would be in order before making any changes to current messaging. In particular, perceptions about climate change may complicate the effectiveness of additional hurricane categories (50).

## 5. Materials and Methods

Observed TC wind speeds are from the International Best Track Archive for Climate Stewardship (*IBTrACS*) and obtainable at https://www.ncdc.noaa.gov/ibtracs/.

To make an attribution statement via Pearl causal inference (21), we calculated PI from climate models with available daily data from the most recent version of the CMIP6. While these models, listed in Table 2, are too coarse (horizontal resolutions >~100 km) to simulate TC wind speed intensities or central pressures, the bulk input fields may be appropriate to an PI calculation. However, we found that CMIP6 model biases were large enough to adversely affect the magnitude of directly simulated PI values. As a result, we bias-corrected all the input fields using the 1979 to 2014 averages from the ERA5 reanalysis. With this correction, the mean values of modeled PI over this period are then well simulated but trends in model PI remain dependent on trends in the corrected input fields. The 1979 to 2014 linear trend in annual category 6 exceedance averaged over the 40S to 10S, 10N to 40N region is shown in the blue histogram of Fig. 1*D* for every realization of the CMIP6 *historical* experiment that provided appropriate fields. The ensemble mean of this histogram is shown by the vertical blue line at 0.16. The red histogram of Fig. 1*D* shows equivalent linear trends over all of the non-overlapping 36-y periods from the CMIP6 pre-industrial (*piControl*) control runs with the ensemble mean shown by the vertical red line at 0.005. The vertical black line at 0.06 shows the 1979 to 2014 ERA5 linear trend averaged over the 40S to 10S, 10N to 40N ocean region. Variability in the *piControl* histogram is controlled by the range of internal variability in the CMIP6 models. Variability in the *historical* histogram is controlled both by internal variability and the range of climate sensitivity in the CMIP6 models. It is important to note that these histograms are a non-uniform “ensemble of opportunity” drawn from different models with varying number of available realizations. A more controlled analysis using large ensemble from multiple models would be preferable but is precluded by the unavailability of the requisite daily model output.

**Table 2.** CMIP6 models used in this study

CMIP6 models used in the PI attribution calculations		CMIP6 models used to perturb the ERA5 under *ssp585* conditions
*historical*	*piControl*	
AWI-ESM-1-1-LR	HadGEM3-GC31-LL	ACCESS-CM2
CNRM-CM6-1	HadGEM3-GC31-MM	ACCESS-ESM1-5
CNRM-ESM2-1	INM-CM4-8	CESM2
CanESM5	MIROC-ES2L	CNRM-CM6-1
GFDL-CM4	UKESM1-0-LL	CanESM5
GFDL-ESM4		FGOALS-g3
HadGEM3-GC31-LL		GFDL-CM4
HadGEM3-GC31-MM		GFDL-ESM4
INM-CM4-8		GISS-E2-1-G
INM-CM5-0		HadGEM3-GC31-LL
MIROC-ES2L		IPSL-CM6A-LR
MIROC6		MIROC6
MPI-ESM1-2-HR		MRI-ESM2-0
MRI-ESM2-0		NorESM2-LM
NorESM2-LM		
NorESM2-MM		
UKESM1-0-LL		

The ERA5 reanalysis fields were obtained from the European Centre for Medium Range Weather Forecasting at https://www.ecmwf.int/en/forecasts/datasets/reanalysis-datasets/era5.

The CMIP6 model data were from the *historical, piControl, hist-nat,* and *ssp585* simulations of the 6th CMIP and downloaded from the Federated Earth System Grid (ESF) at https://esgf-node.llnl.gov/search/cmip6/. Times to global warming levels were prepared by Mathias Hauser (ETH) and are available at https://github.com/mathause/cmip_warming_levels.

PI was calculated using Kerry Emanuel’s Fortran code without modification from daily ERA5 and CMIP6 input data. The method is described at https://emanuel.mit.edu/limits-hurricane-intensity and the code can be downloaded from ftp://texmex.mit.edu/pub/emanuel/TCMAX/.

The bias correction factors of the daily CMIP6 temperature and pressure fields (ta, ts, psl) to the PI code used in Fig. 2 were calculated as a difference between the monthly values of the models and the ERA5 reanalysis averaged over the 1979 to 2014 period. The bias corrections of the daily CMIP6 specific humidity (hus) were calculated as the ratio of the models to the reanalysis. If daily surface temperature (ts) was not available from a CMIP6 model, daily surface air temperature (tas) was substituted for it. Daily bias corrections were linearly interpolated for each day between months. The CMIP6 models with available data for the PI attribution calculation are listed in the first column of Table 2.

The CMIP6 models used to perturb the ERA5 at future global warming levels as shown in Fig. 3 were drawn from the *ssp585* experiment and are listed in the second column of Table 2. Perturbations from pairs of individual realizations of the *historical* and *ssp585* experiments were calculated similar to the bias correction factors and averaged at each model’s native resolution. These individual model average perturbations were then regridded to the coarsest model grid (CanESM2), averaged across models, then regridded again to the finer ERA5 grid.

The finite volume version of Community Atmospheric Model (fvCAM5) developed by the National Center for Atmospheric Research (USA), at a horizontal resolution of about 25 km closely reproduces the observed global frequency of tropical storms over all categories and about half of the observed global frequency of intense TCs (32). The lowest modeled central pressures compare well with the lowest observations, but the corresponding maximum wind speeds are about 5 m/s lower in simulations of the recent past (32). fvCAM5 TC statistics were calculated from 3 hourly output using the Toolkit for Extreme Climate Analysis (51). fvCAM5 output can be downloaded from https://portal.nersc.gov/c20c/data.html.

The MRI-AGCM3.2 model from Japan’s Meteorological Research Institute is the only participant in the CMIP6 High Resolution subproject (HighResMIP) that produced category 5 storms (31, 52). This model, at a horizontal resolution of about 20 km, also well reproduces the observed historical tropical storm frequency and maximum wind speed/minimum central pressure relationship. MRI-AGCM3.2 results were inferred from figure 7 of Murakami et al. (52).

The HiFLOR model developed at the Geophysical Fluid Dynamics Laboratory (USA) is a nudged coupled ocean-atmosphere model. At a horizontal resolution of about 25 km, it simulates about 18% more tropical storms than observed and with higher maximum wind speeds than the other two models considered here. HiFLOR results were inferred from figure 11 of Bhatia et al. (53).

## Data Availability

Data and software used in this paper may be found at https://portal.nersc.gov/cascade/cat6/ (54).

## Footnotes

^*^https://www.ncei.noaa.gov/access/metadata/landing-page/bin/iso?id=gov.noaa.ncdc:C00778.
